# Rapid Expansion of Highly Pathogenic Avian Influenza A(H5N1) Clade 2.3.4.4b Genotype D1.1 Virus across Flyway Regions, North America, Fall 2024

**DOI:** 10.3201/eid3208.260205

**Published:** 2026-08

**Authors:** Matthew Scotch, Temitope O.C. Faleye, Angelica Urquidez-Negrete, Arvind Varsani, Ann Fan, Anne Justice-Allen

**Affiliations:** Arizona State University, Tempe, Arizona, USA (M. Scotch, T.O.C. Faleye, A. Urquidez-Negrete, A. Varsani); Arizona Game & Fish Department, Phoenix, Arizona, USA (A. Fan, A. Justice-Allen)

**Keywords:** Influenza, respiratory infections, viruses, zoonoses, HPAI, influenza A virus, H5N1 subtype, high-throughput nucleotide sequencing, disease transmission, molecular epidemiology, birds, North America

## Abstract

Highly pathogenic avian influenza clade 2.3.4.4b virus continues to circulate in North America and has caused severe human disease. That clade includes genotype D1.1, which became dominant in birds in late 2024. Recent phylodynamic reconstructions place D1.1 emergence in mid-2024 but differ on its inferred origin and early dissemination pathways. We combined targeted surveillance of wild birds in Arizona with publicly available US clade 2.3.4.4b hemagglutinin sequences to estimate when D1.1 genotype emerged and to infer its diffusion among the 4 major US flyways. Phylodynamic analyses showed transitions concentrated among adjacent flyways regions, consistent with stepwise dissemination during fall 2024 and limited support for long-distance Pacific–Atlantic exchange. The Pacific Flyway showed patterns consistent with an early source and the Central Flyway with a secondary hub linked to onward spread. Our findings support coordinated genomic surveillance across adjacent flyways to reduce detection delays and improve situational awareness during rapid viral expansion.

Highly pathogenic avian influenza (HPAI) A(H5N1) clade 2.3.4.4b virus was first detected in North America in December 2021 in the Atlantic Flyway (*1*,*2*). The virus has extensively infected wild birds (*3*), poultry flocks (*4*), cattle (*5*), and numerous mammalian species (*6*). Among various emerging genotypes, D1.1 rapidly became dominant after its detection in the Pacific Flyway in fall 2024. Predominantly found in avian hosts, the D1.1 genotype remained the most abundant lineage for much of 2025 (*7*), accounting for most 2.3.4.4b infections in wild birds and domestic poultry. In addition, D1.1 genotype has exhibited substantial zoonotic potential, evidenced by several confirmed human infections in the United States, including a fatal case in Louisiana (*8*).

Recent phylodynamic reconstructions have begun to resolve the timing of D1.1 virus emergence (*9*; A. Crespo-Bellido et al., unpub. data, https://www.biorxiv.org/content/10.64898/2025.12.19.695329v2), but its early diffusion across the 4 North American Flyway regions remains unresolved. To address that gap, we combined targeted wild bird surveillance in Arizona, within the Pacific Flyway, with phylodynamic analyses of newly generated and publicly available clade 2.3.4.4b hemagglutinin (HA) sequences to reconstruct the timing of early D1.1 virus expansion and infer flyway-scale diffusion patterns. 

## Materials and Methods

Beginning in September 2023, Arizona Game and Fish Department (AZGFD) staff collected cloacal and oropharyngeal swab samples from sick or dead birds as part of routine surveillance and diagnostic testing. We screened for H5 virus via quantitative reverse transcription PCR, performed long-read sequencing, and assembled raw reads via a bioinformatics pipeline (Appendix).

We estimated evolutionary rates and the timing of D1.1 expansion from 660 US avian influenza A(H5N1) clade 2.3.4.4b D1.1 virus genomes by using concatenated whole genomes and individual HA sequences available from GISAID on June 25, 2025 (*7*). We leveraged ModelTest-NG version 0.1.7 (*10*,*11*), which selected general time-reversible plus invariable site plus gamma distribution (GTR + I + Γ) (*12*) as the best fitting DNA substitution model under the Bayesian information criterion (BIC), Akaike information criterion (AIC), and corrected AIC (AICc) metrics for our concatenated genomes, and GTR + Γ under AIC for HA alone. However, the best-fitting root option in TempEST version 1.5.3 (*13*) showed that the concatenated sequences exhibited a positive relationship between sampling time and root-to-tip divergence (correlation coefficient = 0.75), consistent with a clock-like signal, whereas the HA-only sequences exhibited a substantially weaker relationship (correlation coefficient = 0.29).

We implemented Bayesian phylogenetic inference under constant and exponential population growth priors and used BEAST version 10.5.0 (*14*) to run 2 independent Markov chain Monte Carlo (MCMC) methods for 4 × 10^8^ steps, sampling every 10^4^ steps. We used Tracer version 1.7 (*15*) to check model convergence and ensure parameter effective sampling sizes were >200. We evaluated the fit of the constant and exponential growth models to the data by estimating the log marginal likelihoods via stepping-stone and path sampling methods (*16*).

To clarify D1.1 evolutionary diffusion and transmission, we sampled 2.3.4.4b virus HA sequences available from GISAID on April 19, 2026. We selected records with complete collection dates, US state names, and avian hosts collected during June 2024–March 2025. To examine diffusion among wild birds, and because of the large sampling imbalance with domestic poultry, we removed all records with Galliformes as host species. We retained only D1.1 genotype HA sequences. We used the filter module in augur (*17*) to balance our dataset and included 75 sequences per flyway region (n = 300).

We aligned sequences via MAFFT (*18*) and used ModelTest-NG version 0.1.7 (*10*,*11*), which identified GTR + Γ as the best fitting DNA substitution model under the AIC and AICc metrics, and a transition model (TIM1) + Γ under AIC. We used TempEST version 1.5.3 (*13*) and specified the best-fitting root for selecting the appropriate molecular clock for our heterochronous sequences given an augur’s tree function in IQ-TREE (*19*,*20*). Although the weak relationship between root-to-tip divergence and sequence sampling date (correlation coefficient = 0.26) initially suggested the use of a relaxed molecular clock, the posterior mass of the coefficient of variation from preliminary Bayesian MCMC analyses in BEAST version 10.5.0 (*14*) was concentrated near 0, supporting the use of a strict clock.

In BEAUti version 10.5.0 (*14*), we specified a GTR + Γ (*21*) model of nucleotide substitution and considered both constant (*22*) and exponential growth (*23*) coalescent-based tree priors under a strict molecular clock. We ran 2 separate MCMC simulations for 10^8^ steps, sampling every 10^4^ steps, then used Tracer version 1.7 (*15*) and LogCombiner version 10.5.0 (*14*) for model convergence and to combine posterior log files. We evaluated the fit of those 2 models to the data by estimating the log marginal likelihoods via stepping-stone and path sampling methods (*16*).

We used our posterior sample of trees as empirical data for our phylodynamic model to estimate the evolution and transmission of D1.1 virus across North American Flyway regions. Here, for each taxa, we converted state names into respective flyway regions (*24*) and specified a Bayesian stochastic search variable selection (BSSVS) procedure (*25*) with an asymmetric transmission rate matrix. We recorded Markov jumps (*26*) to capture transitions between flyway regions, and recorded Markov rewards to quantify the amount of time lineages spent within each region. We ran 2 separate MCMC simulations for 10^7^ steps, sampling every 10^3^ steps. We extracted Markov jumps with TreeMarkovJumpHistoryAnalyzer in BEAST version 1.10.5 and Markov rewards with TimeSlicer in BEAST version 1.10.4 (*27*). We measured the support for each pairwise transmission in our rate matrix by calculating the Bayes factor (BF) via the SpreaD3 program (*28*) and used a threshold of BF >3 and posterior probability >0.90 to identify the most parsimonious transmission routes.

To clarify the role of early introductions and expansion of D1.1 virus across North American flyways, we used the PersistenceSummarizer module in BEASTX version 10.5 (*29*) to analyze our posterior distribution of Markov jumps from the nearly 3-month period of the initial D1.1 expansion in June 2024 through its first detection in September 2024. The module requires 2 time point specifications: evaluation time (Te) when summarizing state-specific lineages and their descendants after Te and ancestral time (Ta) to indicate the period of lineage persistence up until Te (*30*). We performed logit transformation on the proportion of new introductions, which we defined as lineages that changed flyway states between Ta and Te, and on the proportion of their descendants sampled after Te. We evaluated the relationship between those variables by using a linear regression model, with the logit transformed proportion of sampled descendants after Te arising from those introductions as the response variable. Those calculations enabled us to examine how strongly early introductions influenced D1.1 virus transmission across flyway regions. Finally, we used Bayesian analysis of tip significance (BaTS) (*31*) on the posterior distribution of trees to test whether the observed distribution of flyway traits was significantly associated with phylogenetic structure rather than expected by chance alone.

## Results

**Figure 1:**
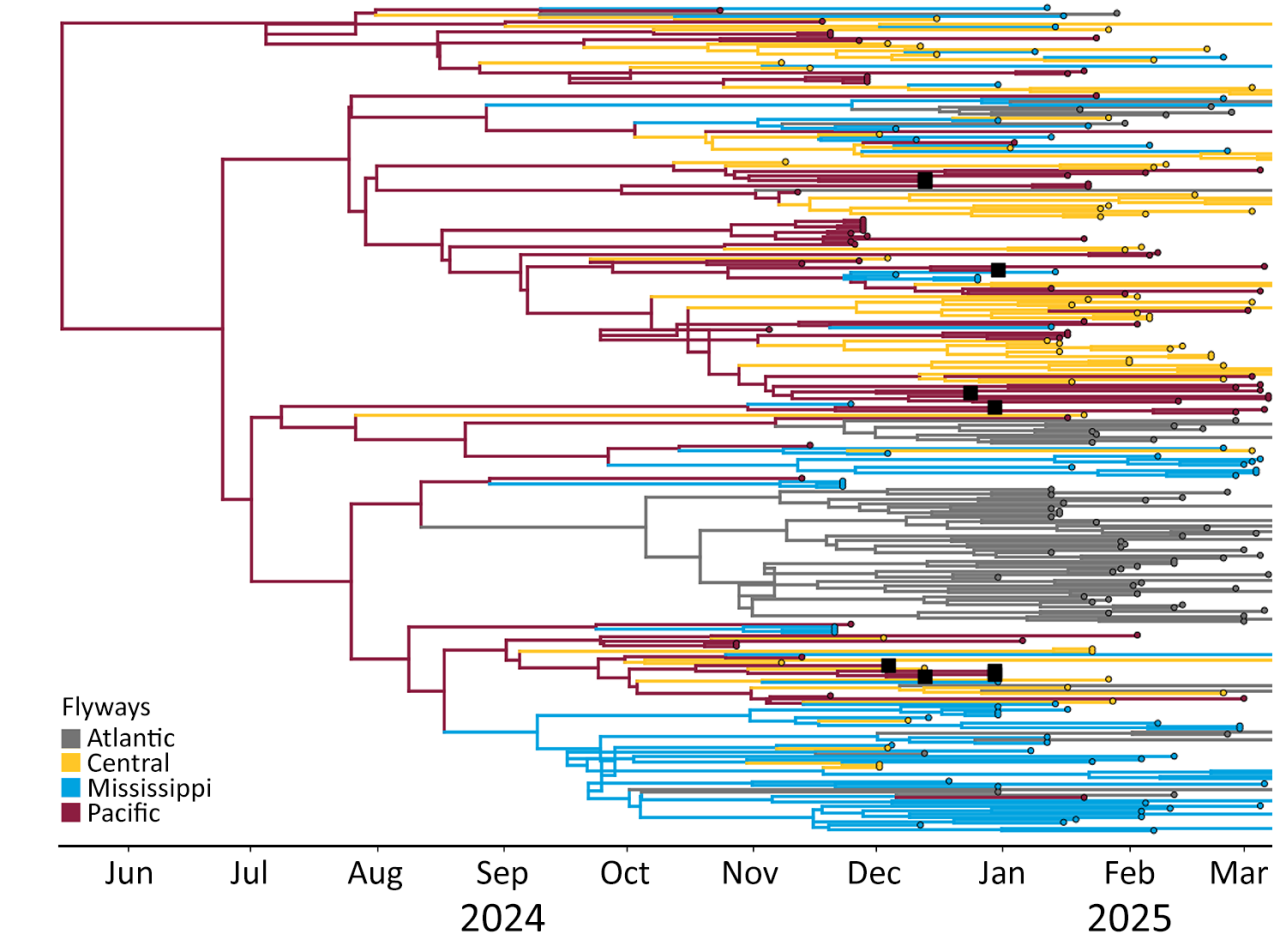
Maximum clade credibility tree of highly pathogenic avian influenza A(H5N1) clade 2.3.4.4b genotype D1.1 virus across flyway regions, North America, fall 2024. Branch colors represent the flyways with the highest posterior probability. The tree represents genotype among avian hosts from 300 taxa (excluding Galliformes) collected during October 2024–March 2025.

During September 2023–May 2025, we tested 177 wild birds from 38 species across 10 of 15 counties in Arizona and identified 10 (6%) HPAI-positive cases (Appendix Table 1, Figure 1). We detected multiple infections among certain species: 3 great horned owls (*Bubo virginianus*), 2 American barn owls (*Tyto furcata*), and 2 red-tailed hawks (*Buteo jamaicensis*).

All but 1 (identification no. 24-0642) HA gene sequences had sufficient coverage depth (>50×); thus, downstream analyses included 9 sequences (Appendix Table 2). We identified the amino acid substitutions and pairwise identity of our highly similar HA sequences (Appendix Figures 2, 3). Not surprisingly, the HA1 subunit, which includes the globular head, receptor binding sites, and major antigenic sites, included the most substitutions (n = 6) compared with the precursor (n = 1) and HA2 (n = 2). No samples contained the Q226L substitution, which has been shown to switch binding specificity from avian to human sialic acid receptors (*32*,*33*).

GenoFlu (https://github.com/USDA-VS/GenoFLU) requires high-confidence matches across all 8 gene segments to assign an overall genotype. Because our amplification assay prioritized HA and neuraminidase (NA) before high-throughput sequencing (Appendix), polymerase segment coverage was incomplete, and GenoFlu could not assign a complete genotype for any of our Arizona isolates (Appendix Table 3). However, across the segments with available lineage calls (HA, NA, nucleoprotein, nonstructural protein, matrix protein, and partial polymerase calls for some isolates), the lineage constellation was consistent with the D1.1 genotype and related D1.X genotypes that share the Eurasian A3 HA lineage (*34*; J.E. Pekar et al., unpub. data, https://virological.org/t/timing-and-molecular-characterisation-of-the-transmission-to-cattle-of-h5n1-influenza-a-virus-genotype-d1-1-clade-2-3-4-4b/991). In addition, HA phylogenetic analysis grouped all our Arizona isolates within the Eurasian A3 HA clade alongside publicly available D1.1 HA sequences (Appendix Figure 4). However, polymerase segments are required for definitive D1.X classification of our isolates.

### Bayesian Inference and Evolutionary Rate Estimates

We used an exponential population growth prior (Appendix Table 4) and estimated the median time to most recent common ancestor (tMRCA) of D1.1 divergence from A3 to be 1.39 (95% highest posterior density [95% HPD] 1.33–1.46) years. That time equates to an estimated date of November 25, 2023 (95% HPD October 30–December 16, 2023). The initial detection date for the Eurasian lineage A3 genotype was April 2022 according to GenoFlu, whereas the estimated TMRCA of A-lineage viruses was August 2021 (*35*), suggesting that A3 evolved and reassorted for multiple months before giving rise to the D1.1 genotype.

We estimated the time of D1.1 virus expansion, its equivalent calendar date, and our evolutionary rate for our HA-only and concatenated sequences (Table 1). We estimated the median HA D1.1–specific tMRCA, equivalent to the time of initial D1.1 expansion, to be 0.80 (95% HPD 0.69–0.94) years, equating to June 27, 2024 (95% HPD May 8–August 5, 2024). We subsequently estimated the median concatenated D1.1-specific tMRCA to be 0.74 (95% HPD 0.69–0.81) years, corresponding to July 19, 2024 (95% HPD June 25–August 6, 2024), which overlaps with other published estimates (*8*). Combined, those estimates are consistent with a period of cryptic (unsampled) circulation between divergence from A3 and subsequent expansion of D1.1 virus that likely spans several months on the basis of the Bayesian HPD intervals.

**Table 1:** Timing of expansion of highly pathogenic avian influenza A(H5N1) clade 2.3.4.4b genotype D1.1 virus across flyway regions, North America, fall 2024*

Dataset	tMRCA, y (95% HPD)	Date (range)	Evolution rate, × 10^−3^ substitutions/site/year (95% HPD)
Hemagglutinin	0.80 (0.69–0.94)	2024 Jun 27 (2024 May 8–Aug 5)	7.16 (6.59–7.75)
Concatenated	0.74 (0.69–0.81)	2024 Jul 19 (2024 Jun 25–Aug 6)	5.56 (5.06–6.04)

*Timing calculated from hemagglutinin and concatenated datasets. Both datasets had the same 660 taxa from October 2024–April 2025. HPD, highest posterior density; tMRCA, time to most recent common ancestor.

Finally, we found that median HA D1.1 evolution rate was 7.16 × 10^−3^ (95% HPD 6.59–7.75 × 10^−3^). For the concatenated dataset, we estimated a median rate of evolution to be 5.56 × 10^−3^ (95% HPD 5.06–6.04 × 10^−3^). That rate is consistent with the finding that the HA segment in D1.1 evolved at a faster rate than earlier non-D1.1 2.3.4.4b HA segments, while the concatenated genome is slower than previous estimates for avian-only taxa (*35*).

### Phylodynamics of D1.1 Genotype across North American Flyways

We generated a maximum clade credibility tree (Figure 1), which inferred the Pacific Flyway as the ancestral state at the root (posterior probability = 0.90). We observed that the Pacific Flyway was predominantly labeled along early inferred branches before transitions to other flyway regions appear later in the tree (Figure 1). The phylogeny then split into 2 major clades with contrasting flyway composition. In the upper clade, we observed a predominance of the Pacific and Central flyways, and occasional transitions involving the Mississippi Flyway. That western clade contrasts with the lower and more eastern clade, where we observed a substantial number of Mississippi and Atlantic flyway branches. Our BaTS results corroborate that visual structure, where we detected significant phylogenetic association with our flyway trait for both the association index and parsimony score (p<0.05) (Table 2). Those findings indicate that D1.1 or D1.1-like HA sequences from the same flyway region were more closely related than expected under random mixing.

**Table 2:** Assessment of phylogenetic structure by flyway region in a study of expansion of highly pathogenic avian influenza A(H5N1) clade 2.3.4.4b genotype D1.1 virus across flyway regions, North America, fall 2024*

Metric	Mean (95% CI)		p value
	Observed	Null	
Associated index	8.32 (6.98–9.57)	26.03 (24.62–27.76)	0
Parsimony score	72.69 (68.0–77.0)	166.08 (160.98–171.33)	0

*Phylogenetic structure by flyway region inferred by BaTS on our posterior set of 18,000 phylodynamic trees from 300 taxa. We ran the simulation using 100 null replicates. The analysis represents 300 HA sequences collected from October 2024–March 2025. BaTS, Bayesian analysis of tip significance.

**Figure 2:**
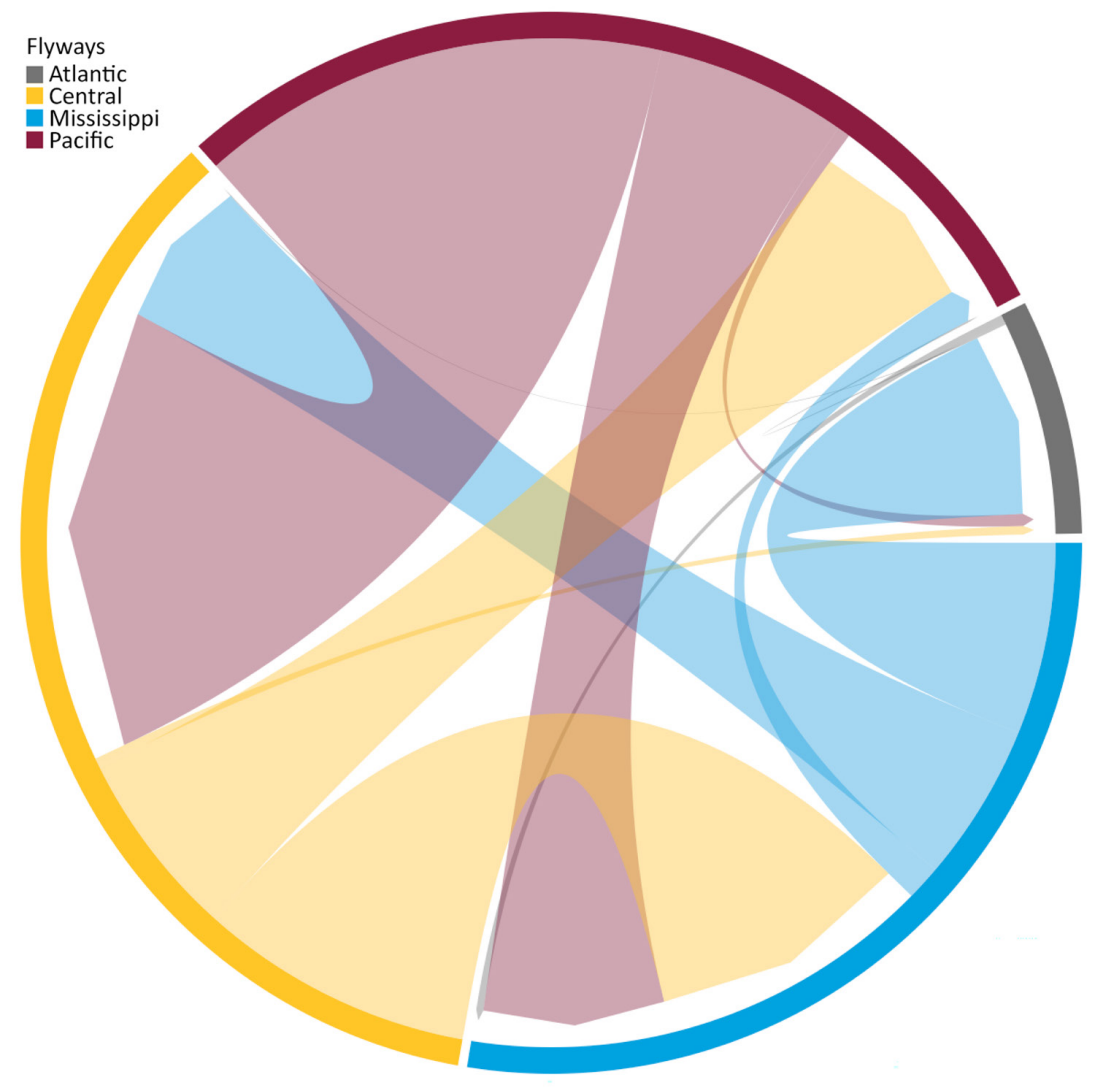
Chord diagram of highly pathogenic avian influenza A(H5N1) clade 2.3.4.4b genotype D1.1 virus across flyway regions, North America, fall 2024. The diagram shows the magnitude of transitions between flyway regions on the basis of the recorded Markov jumps from the posterior distribution of phylodynamic trees. The diagram represents sequences from 300 taxa collected during October 2024–March 2025.

**Figure 3:**
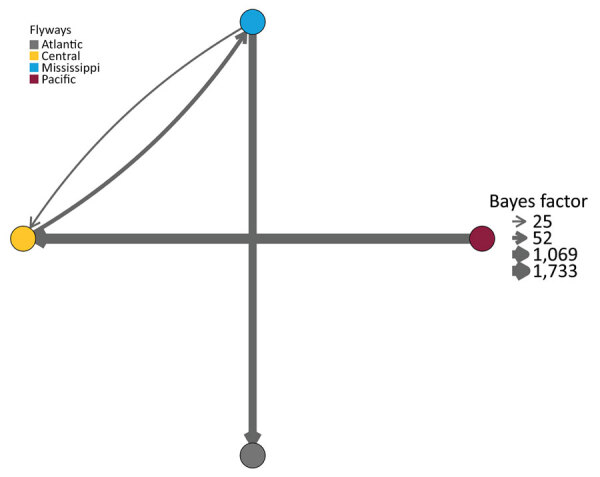
Most parsimonious pairwise transmission routes from a study of expansion of highly pathogenic avian influenza A(H5N1) clade 2.3.4.4b genotype D1.1 virus across flyway regions, North America, fall 2024. Pairs were indicated by Bayes factor results from SpreaD3 (*27*). Routes with a Bayes factor >3 and posterior probability >0.90 are shown. A thicker arrow indicates greater Bayes factor; node colors represent each discrete flyway. The analysis leveraged sequences from 300 taxa collected during October 2024–March 2025.

Posterior Markov jump counts and Bayes factor support for the parsimonious set of nonzero transition rates identified by asymmetric BSSVS (Figure 2, 3; Appendix Table 5) indicated that the best-supported transitions occurred primarily between adjacent flyway regions.Those results are consistent with a predominantly west-to-east dissemination pattern during fall 2024 (e.g., Pacific→Central; Mississippi→Atlantic), while still showing bidirectional exchange among neighboring regions.

**Figure 4:**
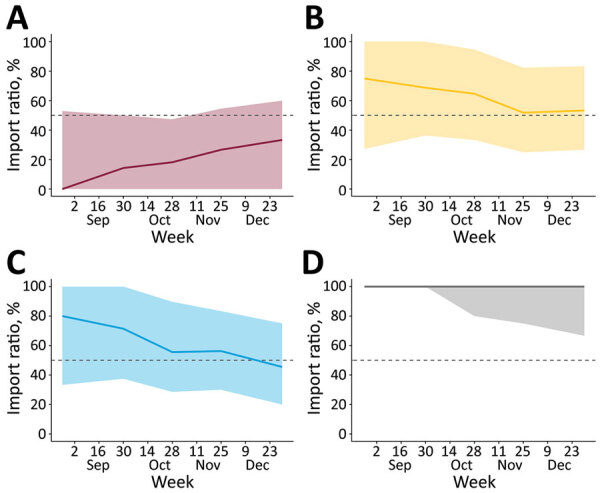
Median ratio of importations to total viral flow from a study of expansion of highly pathogenic avian influenza A(H5N1) clade 2.3.4.4b genotype D1.1 virus across flyway regions, North America, fall 2024. A) Pacific Flyway; B) Central Flyway; C) Mississippi Flyway; D) Atlantic Flyway. Ratios for each flyway were determined by the posterior history of Markov jumps. We show data from September (month of first D1.1 detection) to December of 2025. Values <50% (represented by the dashed line) suggest that a flyway is exporting more than importing (a source flyway), whereas values >50% indicate that a flyway is importing more than exporting (a sink flyway). The shaded areas represented 95% highest posterior density regions. Of note, the posterior history of Markov jumps extends well beyond the timeline and becomes sparse. The analysis leveraged sequences from 300 taxa collected from October 2024–March 2025.

We inferred source–sink dynamics among flyway regions by plotting the ratio of introductions into a flyway to total viral flow involving that flyway for each week on the basis of posterior Markov jumps (Figure 4). A median ratio <50% indicates that a flyway functioned more as a source during that interval, whereas a median >50% indicates it functioned more as a sink. Across the fall months, the Pacific Flyway exhibited median ratios <50% that increased gradually over time, consistent with a predominantly source-like role early in the season. In contrast, the Central Flyway began with ratios >50%, consistent with introduction receipt early in the season, and then trended downward toward 50% later in the fall, suggesting a shift toward a more balanced source–sink profile. The Mississippi Flyway showed median ratios >50% through much of early fall and then decreased toward, and in later weeks approached or breached, 50%, consistent with increasing onward export later in the season when it experienced a surge in cases (Appendix Figure 5). The Atlantic Flyway showed median ratios near 100% across the fall months, suggesting it primarily acted as a sink, where introduced lineages experienced transmission bottlenecks. We also found limited evidence of westward export from the Atlantic region. In addition, Markov reward time estimates further indicated that sustained inferred lineage within flyway regions shifted over time and showed decreasing relative reward time in the Pacific Flyway and increasing reward time in the Mississippi and Atlantic flyways later in fall 2024 (Appendix Figure 6).

**Figure 5:**
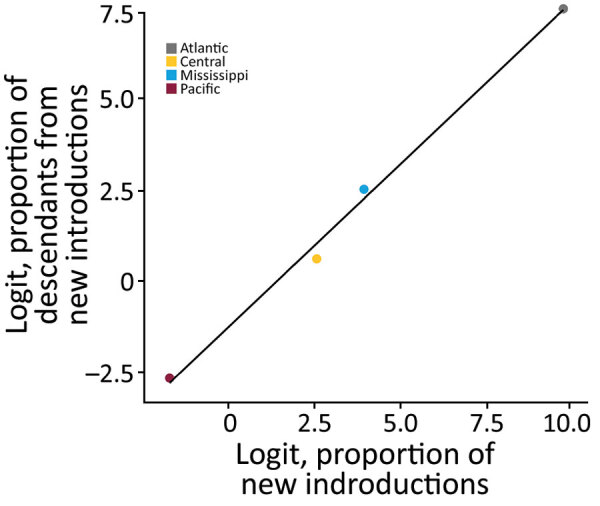
Logit-transformed proportion of new introductions from a study of expansion of highly pathogenic avian influenza A(H5N1) clade 2.3.4.4b genotype D1.1 virus across flyway regions, North America, fall 2024. Graph shows relationship between Ta (estimated D1.1 divergence; June 27, 2024) and Te (approximate date of first D1.1 detection; September 18, 2024) and the logit-transformed proportion of sampled descendant lineages arising from those introductions after Te. Points represent 4 flyway regions of North America. The analysis leveraged sequences from 300 taxa collected from October 2024–March 2025. Ta, ancestral time; Te, evaluation time.

We summarized the early D1.1 genotype expansion window by relating the logit-transformed proportion of inferred new introductions between Ta (estimated D1.1 divergence of June 27, 2024) and Te (approximate date of the first D1.1 detection on September 18, 2024) for each flyway region to the logit-transformed proportion of subsequently sampled descendant lineages arising from introductions after Te (Figure 5). The Pacific Flyway fell in the lower left portion of the plot, indicating a relatively low proportion of new introductions during Ta–Te and a correspondingly low proportion of downstream descendants from those introductions. In contrast, the Atlantic Flyway fell in the upper right portion of the plot, indicating a higher proportion of new introductions during Ta–Te and a higher proportion of downstream descendants after Te. The Central and Mississippi flyways occupy intermediate positions, reflecting more moderate values for both quantities. Because of the small number (n = 4) of flyway regions, we descriptively interpreted that relationship. That early window summary complements the season long import ratio (source–sink) plots by focusing specifically on inferred introductions occurring before, and up to, the initial detection date. The framework provides a concise depiction of inferred introductions during the period of likely cryptic circulation preceding initial detection.

## Discussion

We found a predominantly west-to-east spatial pattern of D1.1 genotype influenza virus dissemination across adjacent North American Flyway regions during fall 2024, with evidence of bidirectional exchange among neighboring regions. Similar North American phylodynamic reconstructions in avian hosts also support rapid dissemination of D1.1 genotype across multiple flyways during fall 2024 (*9*). In this study, we used flyway as a coarse geographic framework for summarizing diffusion rather than as a direct driver of transmission. Those administrative flyway groupings are commonly used in clade 2.3.4.4b virus surveillance reporting in North America (*9*). Accordingly, the flyway-level patterns should be interpreted as broad geographic structure and not direct evidence of mechanisms driving transmission. In addition, the patterns might reflect heterogeneity in surveillance, sampling, and isolation practices. In that context, our findings are consistent with another study (*36*) that found lower migration rates between the most distant flyways (such as between the Pacific and Atlantic flyways) than between neighboring flyways (such as the Central and Mississippi flyways). That finding is also consistent with findings from a study that analyzed earlier clade 2.3.4.4b virus genotypes and observed a distinct east-to-west transmission pattern after introduction into the Atlantic Flyway (L. Damodaran et al., unpub. data, https://www.biorxiv.org/content/10.1101/2024.12.16.628739v2), suggesting that the location of initial introduction can influence inferred directionality across North America. Other work has shown that flyways do not act as strict barriers to gene flow and virus propagation but rather support moderate genetic structuring and cross-flyway transmission (*35*).

In addition to the spatial structuring observed at the scale of flyway regions, we observed temporal shifts in inferred source–sink dynamics and their association with downstream lineage representation. Using an early expansion window (late June to mid-September 2024), flyways with a higher proportion of inferred new introductions also tended to have a higher proportion of subsequently sampled descendant lineages arising from those introductions. Given the small number of flyway regions, we interpret this pattern descriptively as consistent with repeated introductions increasing opportunities for local transmission and subsequent sampling. In our discrete trait phylodynamic analysis, restricted to non-Galliformes avian hosts (Appendix Table 6) and balanced across flyway regions, transitions were concentrated between neighboring flyway regions, with strongest support for Pacific→Central, Central→Mississippi, and Mississippi→Atlantic. In addition, we observed limited evidence for westward export from the Atlantic Flyway during that period. Combined with week-resolved import ratios, our results are consistent with a stepwise expansion in which early circulation in the Pacific Flyway was followed by onward dissemination through the Central and Mississippi flyway regions and into the Atlantic Flyway later in the season, alongside bidirectional exchange among adjacent regions (*9*). Recent North America reconstructions differed in the inferred origin of D1.1 genotype, placing emergence in the Central or possibly Pacific flyway in one analysis (A. Crespo-Bellido et al., unpub. data) and the northern Pacific Flyway in another (*9*).

We estimated that divergence between sampled A3 relatives and the D1.1 HA lineage occurred in late 2023, but our D1.1-specific expansion TMRCA estimates place timing in mid-summer 2024 and overlap independent Bayesian estimates on the basis of concatenated genomes (*9*; A. Crespo-Bellido et al., unpub. data). The period between A3 divergence and D1.1 virus expansion is consistent with unsampled circulation and cryptic transmission observed in earlier 2.3.4.4b virus genotypes (L. Damodaran et al., unpub. data) and with SARS-CoV-2 (*37*,*38*) and could be the result of sampling bias, other circulating strains, or ecologic and geographic factors.

Not surprisingly, our concatenated genomes had a lower nucleotide evolution rate than HA alone but were also lower than previous D1.1 virus genome estimates in avian hosts (J.E. Pekar et al., unpub. data). Despite the high rate of HA evolution, we observed known D1.1 amino acid substitutions, most of which occurred in the HA1 subunit. Fortunately, we did not identify any concerning amino acid substitutions such as Q226L, which has been shown to alter HA binding specificity from avian-preferential α2,3-sialic acid receptors to human-preferential α2,6-sialic acid receptors (*32*,*33*). However, continuous pathogen surveillance remains essential for detecting amino acid substitutions associated with phenotypic changes that might enhance viral fitness, including those that enable human-to-human transmission.

Genotype frequency data from GISAID (*7*) indicate that, as of spring 2026, D1.1 is the dominant 2.3.4.4b virus genotype in the United States after cocirculation with B3.13 for much of 2025 (Appendix Figure 7). Those rapid shifts in genotype dominance underscore the need for continuous genomic surveillance and highlight the risk for rapid viral expansion, illustrated by the fatal human infection in Louisiana, USA, in early 2025 (*8*) and a case of direct transmission from wild birds in British Columbia, Canada (*34*). Finally, our Arizona wildlife surveillance detected D1.1-like viruses in the Pacific Flyway during the inferred expansion period, providing local genomic evidence to support our North American Flyway phylodynamic reconstruction and illustrates how routine sentinel sequencing can support avian influenza epidemiology.

The first limitation of our study is that we used individual HA gene sequences rather than concatenated segments for phylodynamic inference. As the primary surface protein influencing antigenicity and host adaptation, HA is highly informative for influenza A phylodynamics, including clade 2.3.4.4b virus (*39*–*42*). Second, we also used HA to estimate the deeper A3–D1.1 divergence on the basis of its shared Eurasian ancestry. However, our divergence time estimates might be sensitive to the single-gene framework and would likely differ from estimates generated from segment sets. In addition, although we recovered lineage calls for multiple segments, including HA, NP, NA, MP, and NS, polymerase gene coverage was incomplete across our Arizona specimens. Therefore, GenoFlu could not assign a complete genotype, and we cannot definitively distinguish among closely related D1.X genotypes that differ in the polymerase segments basic 2, basic 1, and acidic. Third, we restricted our HA phylodynamic dataset to sequences from the United States. During October 2024–March 2025, ≈80% of available North American D1.1 HA sequences with sufficient metadata on GISAID (as of April 25, 2026) were from US states, whereas ≈20% were from Canada and <1% were from Mexico. Including the comparatively sparse sequences from Canada and Mexico would have introduced additional temporal and spatial sampling heterogeneity with little influence on the diffusion analysis. Therefore, we interpreted our phylodynamics within the US portions of each flyway region. Fourth, our phylodynamic model did not explicitly include avian species abundance, poultry density, climate, or environmental covariates; thus, our inferred flyway region transitions should be interpreted as broad summaries of D1.1 diffusion rather than direct evidence of the mechanisms driving avian influenza spread over these regions. Finally, our Arizona surveillance initiative was opportunistic and focused largely on sick or dead birds, and was not designed to estimate statewide prevalence, species-specific risk, or the timing of first introduction into Arizona.

In conclusion, our results indicate that D1.1 genotype virus likely circulated for months before detection and then disseminated rapidly across flyway regions, highlighting a key surveillance gap that emerging clade 2.3.4.4b virus genotypes could occur and spread before they are identified through routine surveillance. Those findings support earlier, coordinated genomic surveillance, particularly among adjacent flyway regions, in the weeks preceding and during early migration. Such efforts must include use of standardized metadata, rapid sequencing, and validated bioinformatics pipelines to support optimal data sharing and reporting. Linking wildlife, poultry, and mammalian surveillance within a One Health framework and routinely comparing newly generated sequences against existing regional datasets could help detect cryptic circulation sooner and enable earlier situational awareness and response.

Appendix Additional information on rapid expansion of highly pathogenic avian influenza A(H5N1) clade 2.3.4.4b genotype D1.1 virus across flyway regions, North America, fall 2024.
