# Supplementary material for: Rapid Expansion of Highly Pathogenic Avian Influenza A(H5N1) Clade 2.3.4.4b Genotype D1.1 Virus across Flyway Regions, North America, Fall 2024
Source: Emerg Infect Dis. 2026 Aug;32(8):1241–50. doi: 10.3201/eid3208.260205 (PMC13426862; doi:10.3201/eid3208.260205)
Supplement: Appendix — Additional information on rapid expansion of highly pathogenic avian influenza A(H5N1) clade 2.3.4.4b genotype D1.1 virus across flyway regions, North America, fall 2024. [file 26-0205-Techapp-s1.pdf]

Article DOI: https://doi.org/10.3201/eid3208.260205

EID cannot ensure accessibility for supplementary materials supplied by authors. Readers who have difficulty accessing supplementary content should contact the authors for assistance.

Rapid Expansion of Highly Pathogenic Avian Influenza A(H5N1) Clade 2.3.4.4b Genotype D1.1 Virus across Flyway Regions, North America, Fall 2024

Appendix

Methods

HPAI sampling among wild birds

Arizona Game and Fish Department (AZGFD) staff collected cloacal and oropharyngeal swab samples from sick or dead birds from 25 September 2023 to 23 May 2025, as part of routine surveillance and diagnostic testing for symptomatic birds. We did not include high-priority samples associated with significant mortality events or cases with known human exposure, as these samples were tested at the Arizona Veterinary Diagnostic Laboratory. We also excluded samples from hunter-harvested birds. For each bird, AZGFD staff collected separate cloacal and oropharyngeal swab samples in universal transport media. There were occasional instances in which staff collected only cloacal or oropharyngeal swabs, or swabs from alternative sites such as the trachea, due to carcass condition. AZGFD personnel also used a standardized template to record the date of collection, the bird species, the location, and the health status, including symptoms and whether it was alive or deceased. The study team then entered all recorded information into a REDCap database at Arizona State University (ASU).

Molecular detection and sequencing

We performed all laboratory analysis at the Biodesign Institute at Arizona State University (ASU), Tempe, AZ in a BSL 2+ laboratory. If not processed immediately after transport, we stored samples at −80°C. We pooled equal volumes of cloacal and oropharyngeal swabs and subjected 140°µl of the pooled sample to RNA extraction using the QIAamp Viral

RNA kit according to the manufacturer's specification (Qiagen, Inc., Venlo, Netherlands). We tested each extract for influenza A virus using a reverse transcription real-time polymerase chain reaction (PCR) assay as previously described (*1*) and subjected samples with a cycle threshold (Ct) value <38 to cDNA synthesis using the uniflu primer as previously described (*1*). We amplified the hemagglutinin (HA) and neuraminidase (NA) gene segments using the protocol described by Zhou *et al.* (*2*) and previously outlined (*3*), followed by purification of the amplicons with magnetic beads and library preparation using kits SQK-LSK110 and EXP-NBD104. For long-read sequencing, we used a Flongle flow cell (R10.4.1) on a MinION Mk1b device (Oxford Nanopore Technologies, Oxford, UK) as previously described (*1*). We used Guppy for basecalling, which is implemented within MinKNOW software.

Processing and annotation of A/H5 2.3.4.4b long-read sequences

We used NanoPlot v1.44.1 (*4*) to assess the quality of our long read sequencies and trimmed adapters from 5′ and 3′ ends using Porechop v0.2.4 (*5*). To remove low-quality reads, we applied Fastplong v0.2.2 (*6*), excluding those with a mean Q score below 10 or length under 500 nt. For downstream processing, we shortened FASTQ headers using Seqtk v1.5 (*7*).

For our analysis, we focused on the hemagglutinin (HA) gene due to its high rate of nucleotide evolution compared to other segments, and its frequent use in genomic epidemiology for understanding the evolutionary diffusion of influenza viruses. We used the FLU-minion module in IRMA v1.2.0 (*8*) for influenza reference-based assembly, discarded any HA sequence with coverage depth <50x, and used medaka v1.11.3 (*9*) to polish consensus sequences. We used FLAN (*10*) for segment-specific annotation and examined HA sequence similarity via SDT 2 vBeta 4 (*11*).

HA-based D1.1 lineage assignment and timing of HA divergence from A3

We uploaded our HA sequences to GISAID's BLAST feature (*12*,*13*) which revealed top hits to HA sequences annotated as genotype D1.1 (via GenoFlu (*14*)) in GISAID. In addition, we searched GISAID (*13*) on April 18, 2026, for 2.3.4.4b HA sequences from original specimens collected from avian hosts in the U.S. and Canada with complete dates between December 1, 2021, and March 31, 2025. In Geneious Prime v2026.0.2 (Dotmatics, Boston, Massachusetts, USA), we trimmed each sequence to the start and stop codon and removed any duplicates. These 4,829 sequences spanned A, B, C, and D genotypes as well as less prevalent genotypes classified

as *minor*. We combined this dataset with our 9 sequences, produced a maximum likelihood tree via Nextstrain's augur (*15*), and visualized the arrangement of our Arizona taxa within the phylogenetic tree via auspice.us.

To estimate the timing of D1.1 HA divergence from A3, we combined our nine HA sequences with 701 D1.1 HA segments from GISAID that were available on June 25, 2025. We included sequences with a start and stop codon and those that received a Nextclade (*16*,*17*) quality status of *good* or *mediocre*. We added three A3 HA segments that we determined to be recently divergent via our maximum likelihood tree. ModelTest-NG v0.1.7 (*18*,*19*) identified TIM1 + Γ (BIC metric), GTR + Γ (AIC metric), and K80 (AICc metric) as the DNA substitution models with the best fit given the sequence data. Because two of the three best-supported models were relatively complex, and because TIM1 is a constrained version of GTR with k+6 free parameters (*20*), we selected GTR + Γ (*21*) for this dataset.

We used TempEST v1.5.3 (*22*) for selecting the appropriate molecular clock given our heterochronous sequences given an initial maximum likelihood phylogenetic tree produced by IQ-TREE (*23*) via augur's *tree* function (*24*). We used the *best-fitting roo*t option which showed a correlation coefficient of 0.13, suggesting a weak relationship between sampling time and root-to-tip divergence and the need for a relaxed molecular clock.

For Bayesian phylogenetic inference, we used BEASTv10.5.0 (*25*) with BEAGLE v3 (*26*) under a GTR + Γ (*21*,*27*) model of nucleotide substitution and considered both constant (*28*) and exponential growth (*29*) coalescent-based tree priors with a uncorrelated lognormal relaxed molecular clock (*30*). We evaluated the fit of these population growth models to the data by estimating the log marginal likelihoods via stepping-stone and path sampling methods (*31*). We combined three independent Markov chain Monte Carlo simulations (MCMC) for 4×10^8 steps, sampling every 4×10^4 steps, after assessing model convergence and parameter effective sampling sizes (ESS >200) using Tracer v1.7 (*32*). After combining our log files via LogCombiner v10.5.0, we used Tracer to summarize the time to the most recent ancestor (TMRCA).

References

1. Scotch M, Faleye TOC, Wright JM, Finnerty S, Halden RU, Varsani A. Campus-based genomic surveillance uncovers early emergence of a future dominant A(H3N2) influenza clade. iScience. 2025;28:113941. PubMed https://doi.org/10.1016/j.isci.2025.113941

2. Zhou B, Deng YM, Barnes JR, Sessions OM, Chou TW, Wilson M, et al. Multiplex reverse transcription-PCR for simultaneous surveillance of influenza A and B viruses. J Clin Microbiol. 2017;55:3492–501. PubMed https://doi.org/10.1128/JCM.00957-17

3. Faleye TOC, Adams D, Adhikari S, Sandrolini H, Halden RU, Varsani A, et al. Use of hemagglutinin and neuraminidase amplicon-based high-throughput sequencing with variant analysis to detect co-infection and resolve identical consensus sequences of seasonal influenza in a university setting. BMC Infect Dis. 2021;21:810. PubMed https://doi.org/10.1186/s12879-021-06526-5

4. De Coster W, Rademakers R. NanoPack2: population-scale evaluation of long-read sequencing data. Bioinformatics. 2023;39:btad311. PubMed https://doi.org/10.1093/bioinformatics/btad311

5. Wick R. Porechop. 2018 Aug 5 [cited 2026 Jan 28]. https://github.com/rrwick/Porechop

6. Chen S. Ultrafast one-pass FASTQ data preprocessing, quality control, and deduplication using fastp. iMeta. 2023;2:e107. PubMed https://doi.org/10.1002/imt2.107

7. Li H. Seqtk. 2026 [cited 2026 May 1]. https://github.com/lh3/seqtk

8. Shepard SS, Meno S, Bahl J, Wilson MM, Barnes J, Neuhaus E. Viral deep sequencing needs an adaptive approach: IRMA, the iterative refinement meta-assembler. BMC Genomics. 2016;17:708. PubMed https://doi.org/10.1186/s12864-016-3030-6

9. Oxford Nanopore Technologies. Medaka 2026 [cited 2026 Jan 28]. https://github.com/nanoporetech/medaka

10. Bao Y, Bolotov P, Dernovoy D, Kiryutin B, Tatusova T. FLAN: a web server for influenza virus genome annotation. Nucleic Acids Res. 2007;35:W280–4. PubMed https://doi.org/10.1093/nar/gkm354

11. Muhire BM, Roumagnac P, Varsani A, Martin DP. Sequence Demarcation Tool (SDT), a free user-friendly computer program using pairwise genetic identity calculations to classify nucleotide or amino acid sequences. Methods Mol Biol. 2025;2912:71–9. PubMed https://doi.org/10.1007/978-1-0716-4454-6_9

12. Altschul SF, Gish W, Miller W, Myers EW, Lipman DJ. Basic local alignment search tool. J Mol Biol. 1990;215:403–10. PubMed https://doi.org/10.1016/S0022-2836(05)80360-2

13. Shu Y, McCauley J. GISAID: Global Initiative on Sharing All Influenza Data – from vision to reality. Euro Surveill. 2017;22:30494. PubMed https://doi.org/10.2807/1560-7917.ES.2017.22.13.30494

14. US Department of Agriculture. GenoFLU. 2025 Jun 23 [cited 2026 May 1]. https://github.com/USDA-VS/GenoFLU

15. Hadfield J, Megill C, Bell SM, Huddleston J, Potter B, Callender C, et al. Nextstrain: real-time tracking of pathogen evolution. Bioinformatics. 2018;34:4121–3. PubMed https://doi.org/10.1093/bioinformatics/bty407

16. Aksamentov I, Roemer C, Hodcroft EB, Neher RA. Nextclade: clade assignment, mutation calling and quality control for viral genomes. J Open Source Softw. 2021;6:3773. https://doi.org/10.21105/joss.03773

17. Ort JT, Zolnoski SA, Lam TT, Neher R, Moncla LH. Development of avian influenza A(H5) virus datasets for Nextclade enables rapid and accurate clade assignment. bioRxiv. 2025.

18. Darriba D, Posada D, Kozlov AM, Stamatakis A, Morel B, Flouri T. ModelTest-NG: a new and scalable tool for the selection of DNA and protein evolutionary models. Mol Biol Evol. 2020;37:291–4. PubMed https://doi.org/10.1093/molbev/msz189

19. Flouri T, Izquierdo-Carrasco F, Darriba D, Aberer AJ, Nguyen LT, Minh BQ, et al. The phylogenetic likelihood library. Syst Biol. 2015;64:356–62. PubMed https://doi.org/10.1093/sysbio/syu084

20. Posada D. jModelTest: phylogenetic model averaging. Mol Biol Evol. 2008;25:1253–6. PubMed https://doi.org/10.1093/molbev/msn083

21. Tavaré S. Some probabilistic and statistical problems in the analysis of DNA sequences. In: Miura R, editor. Lectures on Mathematics in the Life Sciences. Providence (RI): American Mathematical Society; 1985. p. 57–86.

22. Rambaut A, Lam TT, Max Carvalho L, Pybus OG. Exploring the temporal structure of heterochronous sequences using TempEst (formerly Path-O-Gen). Virus Evol. 2016;2:vew007. PubMed https://doi.org/10.1093/ve/vew007

23. Minh BQ, Schmidt HA, Chernomor O, Schrempf D, Woodhams MD, von Haeseler A, et al. IQ-TREE 2: new models and efficient methods for phylogenetic inference in the genomic era. Mol Biol Evol. 2020;37:1530–4. PubMed https://doi.org/10.1093/molbev/msaa015

24. Huddleston J, Hadfield J, Sibley TR, Lee J, Fay K, Ilcisin M, et al. Augur: a bioinformatics toolkit for phylogenetic analyses of human pathogens. J Open Source Softw. 2021;6:2906. PubMed https://doi.org/10.21105/joss.02906

25. Suchard MA, Lemey P, Baele G, Ayres DL, Drummond AJ, Rambaut A. Bayesian phylogenetic and phylodynamic data integration using BEAST 1.10. Virus Evol. 2018;4:vey016. PubMed https://doi.org/10.1093/ve/vey016

26. Ayres DL, Cummings MP, Baele G, Darling AE, Lewis PO, Swofford DL, et al. BEAGLE 3: improved performance, scaling, and usability for a high-performance computing library for statistical phylogenetics. Syst Biol. 2019;68:1052–61. PubMed https://doi.org/10.1093/sysbio/syz020

27. Yang Z. Maximum likelihood phylogenetic estimation from DNA sequences with variable rates over sites: approximate methods. J Mol Evol. 1994;39:306–14. PubMed https://doi.org/10.1007/BF00160154

28. Kingman JFC. The coalescent. Stochastic Process Appl. 1982;13:235–48. https://doi.org/10.1016/0304-4149(82)90011-4

29. Griffiths RC, Tavaré S. Sampling theory for neutral alleles in a varying environment. Philos Trans R Soc Lond B Biol Sci. 1994;344:403–10. PubMed https://doi.org/10.1098/rstb.1994.0079

30. Drummond AJ, Ho SY, Phillips MJ, Rambaut A. Relaxed phylogenetics and dating with confidence. PLoS Biol. 2006;4:e88. PubMed https://doi.org/10.1371/journal.pbio.0040088

31. Baele G, Lemey P, Bedford T, Rambaut A, Suchard MA, Alekseyenko AV. Improving the accuracy of demographic and molecular clock model comparison while accommodating phylogenetic uncertainty. Mol Biol Evol. 2012;29:2157–67. PubMed https://doi.org/10.1093/molbev/mss084

32. Rambaut A, Drummond AJ, Xie D, Baele G, Suchard MA. Posterior summarization in Bayesian phylogenetics using Tracer 1.7. Syst Biol. 2018;67:901–4. PubMed https://doi.org/10.1093/sysbio/syy032

33. Bielejec F, Baele G, Vrancken B, Suchard MA, Rambaut A, Lemey P. SpreaD3: interactive visualization of spatiotemporal history and trait evolutionary processes. Mol Biol Evol. 2016;33:2167–9. PubMed https://doi.org/10.1093/molbev/msw082

34. Jeffreys H. Theory of probability, 3rd edition. Oxford (UK): Clarendon Press; 1998.

35. US Fish and Wildlife Service, Natural Resource Program Center. Administrative waterfowl flyway boundaries 2017 [cited 2026 May 1]. https://iris.fws.gov/APPS/ServCat/Reference/Profile/42276

Appendix Table 1. Highly pathogenic avian influenza (HPAI) positive birds identified in this study collected in December 2024 and March 2025

Bird ID	Collection date	Arizona County	Town	Species
24–0514	2024–12–04	Pinal	Florence	*Buteo jamaicensis*
24–0522	2024–12–13	Maricopa	Litchfield Park	*Bubo virginianus*
24–0584	2024–12–13	Maricopa	Scottsdale	Domestic goose *(Anser)*
24–0585	2024–12–13	Maricopa	Scottsdale	*Branta canadensis*
24–0623	2024–12–24	Maricopa	Surprise	*Bubo virginianus*
24–0629	2024–12–30	Pinal	Queen Creek	*Tyto furcata*
24–0630	2024–12–30	Pinal	Casa Grande	*Parabuteo unicinctus*
24–0631	2024–12–30	Maricopa	Queen Creek	*Buteo jamaicensis*
24–0632	2024–12–31	Pinal	San Tan Valley	*Tyto furcata*
24–0642	2025–03–20	Yavapai	Prescott	*Bubo virginianus*

Appendix Table 2. Median coverage depth by segment for each infected bird from avian influenza positive samples collected in December 2024 and March 2025*

	Median Coverage Depth (x)							
Bird ID	PB2	PB1	PA	HA	NP	NA	MP	NS
24–0514	1	154	5	2,438	2,901	2,898	1,418	10,213
24–0522	30	7	3	2,166	3,559	3,128	3,577	10,482
24–0584	2	32	2	3,354	4,244	4,130	3,450	9,955
24–0585	NMR	NMR	4	252	115	551	358	926
24–0623	17	3	6	1,569	1,518	1,787	1,099	5,214
24–0629	NMR	7	1	2,255	2,677	2,799	1,187	2,393
24–0630	NMR	NMR	NMR	1,214	440	339	63	1,103
24–0631	27	NMR	NMR	2,273	2,224	2,155	1,630	2,989
24–0632	NMR	NMR	4	70	65	80	79	264
24–0642	NMR	NMR	NMR	4	NMR	5	11	90

*HA was the focus of this study. NMR, no mapped reads.

Appendix Table 3. GenoFLU results for each influenza gene segment from avian influenza positive samples collected in December 2024*

Bird ID	Genotype	PB2	PB1	PA	HA	NP	NA	MP	NS
24–0514	Not Assigned	?	ea3	am4	ea3	am13	am4N1	ea3	ea3
24–0522	Not Assigned	am24	ea3	?	ea3	am13	am4N1	ea3	ea3
24–0584	Not Assigned	?	ea3		ea3	am13	am4N1	ea3	ea3
24–0585	Not Assigned	?	?	am4	ea3	am13	am4N1	ea3	ea3
24–0623	Not Assigned	am24	?	am4	ea3	am13	am4N1	ea3	ea3
24–0629	Not Assigned	?	ea3	am4	ea3	am13	am4N1	ea3	ea3
24–0630	Not Assigned	?	?	?	ea3	am13	am4N1	ea3	ea3
24–0631	Not Assigned	am24	?	?	ea3	am13	am4N1	ea3	ea3
24–0632	Not Assigned	?	?	am4	ea3	am13	am4N1	ea3	ea3

*We excluded sample 24–0642 collected in March 2025 because of insufficient coverage depth for our analysis. Am, North American lineage; ea, Eurasian lineage; ?, <98.0% match found or segment not present.

Appendix Table 4. Log marginal likelihood results for Bayesian inference models used in this study from avian influenza positive samples collected in December 2024*

Influenza genes	Analyses	No. taxa	Genotype(s)	Tree prior	Stepping-stone	Path-sampling
HA	D1.1 emergence from A3	713	A3 + D1.1	Exponential	−9,779.2	−9,758.5
HA	D1.1 emergence from A3	713	A3 + D1.1	Constant	−9,996.8	−9,985.3
HA	D1.1 expansion	660	D1.1	Exponential	−9,217.5	−9,207.56
HA	D1.1 expansion	660	D1.1	Constant	−9,409.2	−9,396.23
8 concatenated	D1.1 expansion	660	D1.1	Exponential	−61,115.5	−61,090.4
8 concatenated	D1.1 expansion	660	D1.1	Constant	−61,112.7	−61,092.4
HA	D1.1 phylodynamics	300	D1.1	Exponential	−7,695.1	−7,692.9
HA	D1.1 phylodynamics	300	D1.1	Constant	−7,761.3	−7,759.2

*We show estimates for both stepping-stone and path-sampling methods for each of our datasets. We indicate in gray the models with higher likelihood results. For the HA-only datasets, the models with an exponential growth prior had higher likelihood results. Meanwhile, for the concatenated dataset, the likelihoods were mixed for exponential and constant tree priors.

Appendix Table 5. Bayes factor (BF) results for pairwise flyway transmission routes of D1.1 of 300 HA sequences, October 2024–March 2025*

From	To	Bayes Factor	Posterior Probability	Level of Support
Pacific	Central	1,732.9	0.999	Decisive
Mississippi	Atlantic	1,069.5	0.998	Decisive
Central	Mississippi	52.2	0.959	Very Strong
Mississippi	Central	24.8	0.917	Strong
Central	Pacific	16.0	0.877	Strong
Pacific	Mississippi	11.8	0.84	Strong
Mississippi	Pacific	1.6	0.414	Barely Worth Mentioning
Atlantic	Mississippi	1.0	0.312	Barely Worth Mentioning
Pacific	Atlantic	1.0	0.299	Barely Worth Mentioning
Central	Atlantic	0.6	0.223	Barely Worth Mentioning
Atlantic	Pacific	0.4	0.161	Barely Worth Mentioning
Atlantic	Central	0.3	0.114	Barely Worth Mentioning

*BF results generated by SpreaD3 (*33*). We set criteria of BF of >3 and a posterior probability $\geq$0.90 and use a gray line to denote those achieving this threshold. We also include the level of support that is associated with the Bayes factor as defined by Jeffreys (*34*).

Appendix Table 6. Avian host composition of 300 HA D1.1 sequences used in the discrete trait analyses, October 2024–March 2025.

Host order	No. (%) sequences, n = 300
Accipitriformes	128 (42.67)
Anseriformes	110 (36.67)
Strigiformes	29 (9.67)
Charadriiformes	9 (3.00)
Falconiformes	7 (2.33)
Passeriformes	7 (2.33)
Pelecaniformes	5 (1.67)
Phoenicopteriformes	3 (1.00)
Gruiformes	1 (0.33)
Avian (Unknown)	1 (0.33)

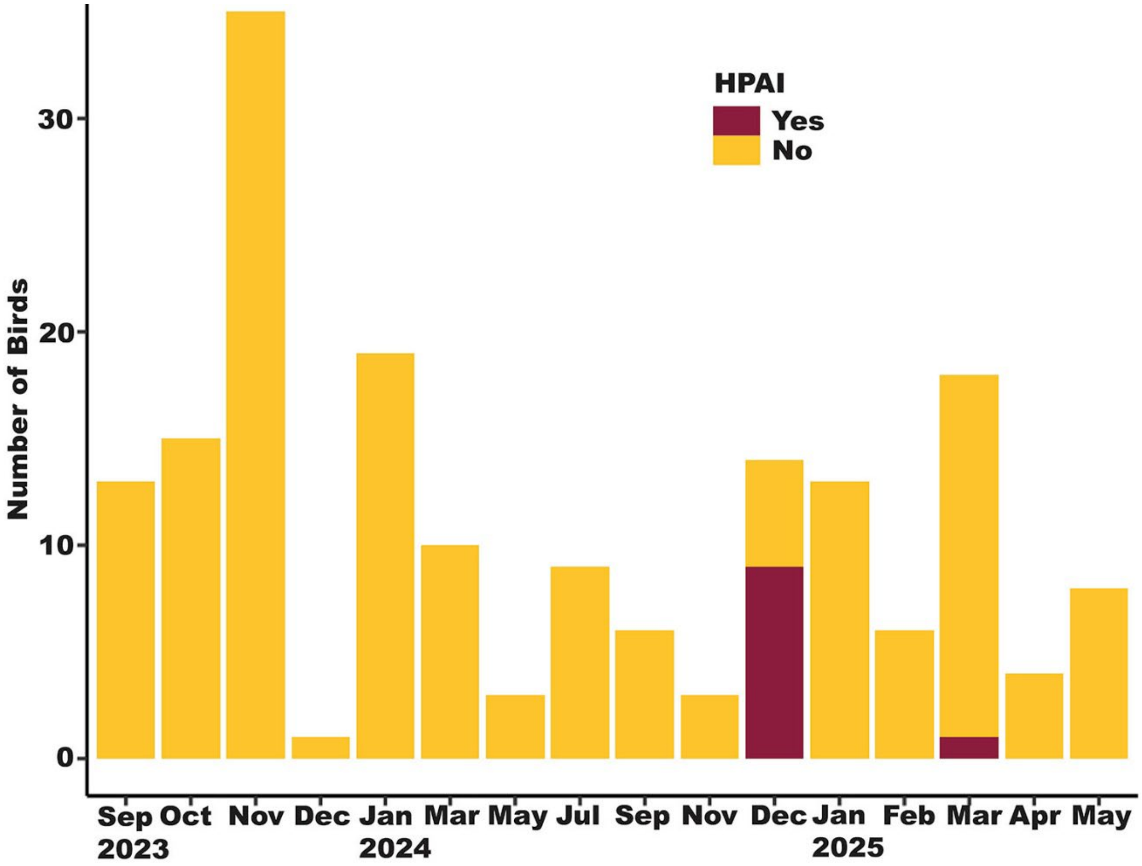

Appendix Figure 1. Number of birds collected in Arizona over time and highly pathogenic avian influenza (HPAI) status for this study, September 2023–May 2025.

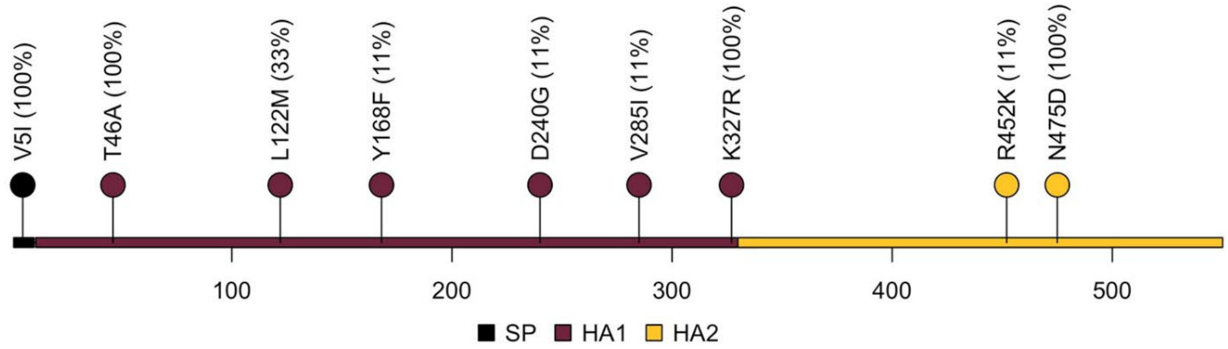

Appendix Figure 2. Lollipop chart showing the observed amino acid substitutions of the nine avian influenza positive samples collected in December 2024 against reference HA A/Astrakhan/3212 cell/2020(H5N8). Percentages indicate the proportion of the nine sequences that contained the substitution (H3 numbering) and colors partition the HA gene segment. Abbreviations: SP - signal peptide.

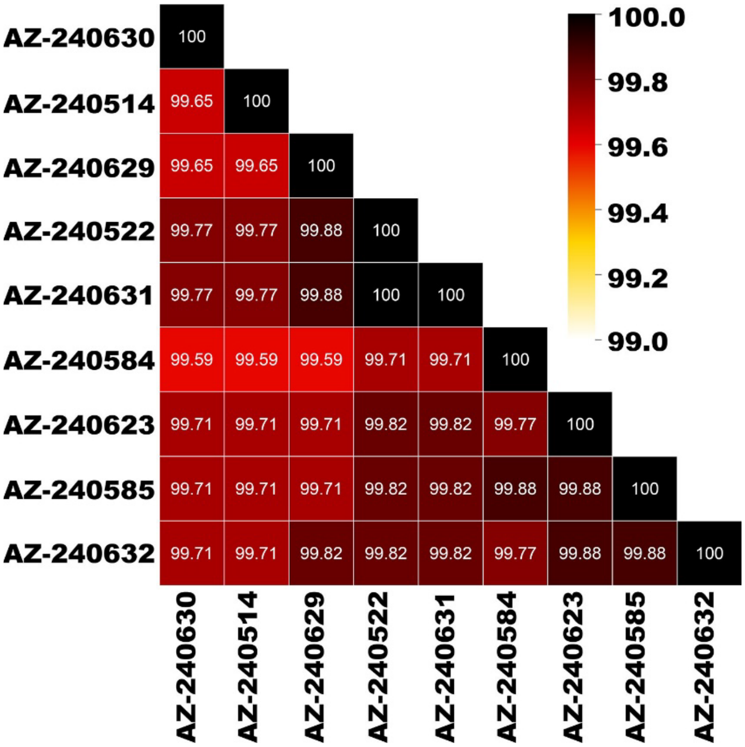

Appendix Figure 3. Sequence similarity results from SDT 2 vBeta 4 (*11*) for the nine D1.1 HA sequences generated from this study collected in December 2024.

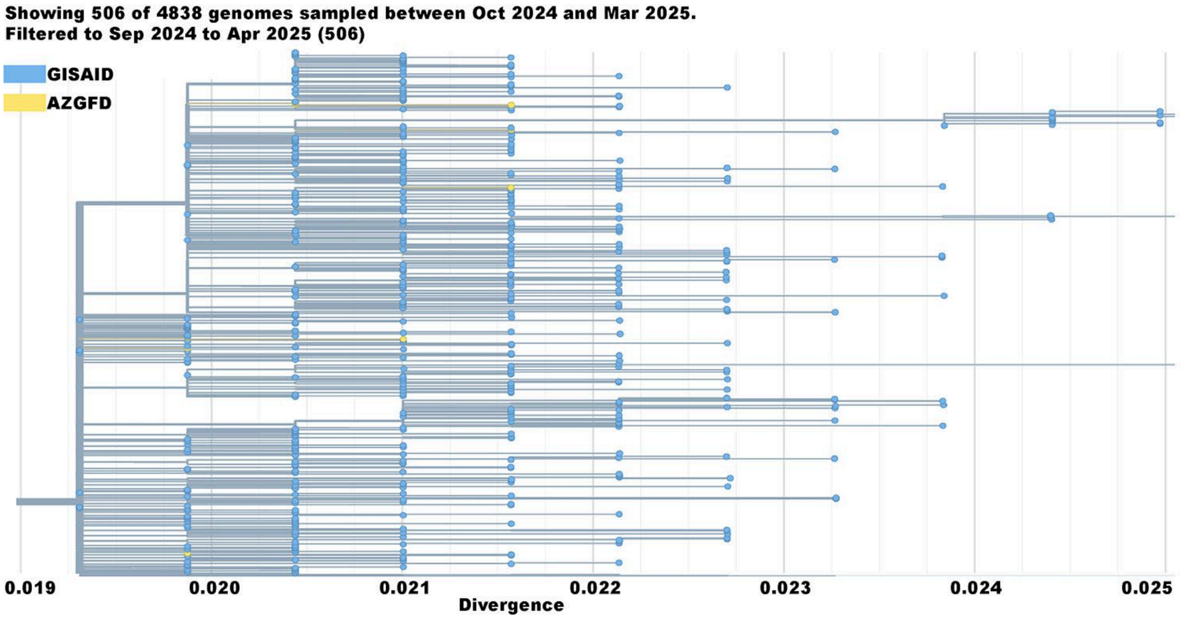

Appendix Figure 4. Nextstrain HA phylogeny including 4,829 GISAID sequences and our 9 new Arizona sequences, December 2021–March 2025. Our Arizona Game & Fish Department (AZGFD) sequences are positioned in a D.1.1-dominant clade.

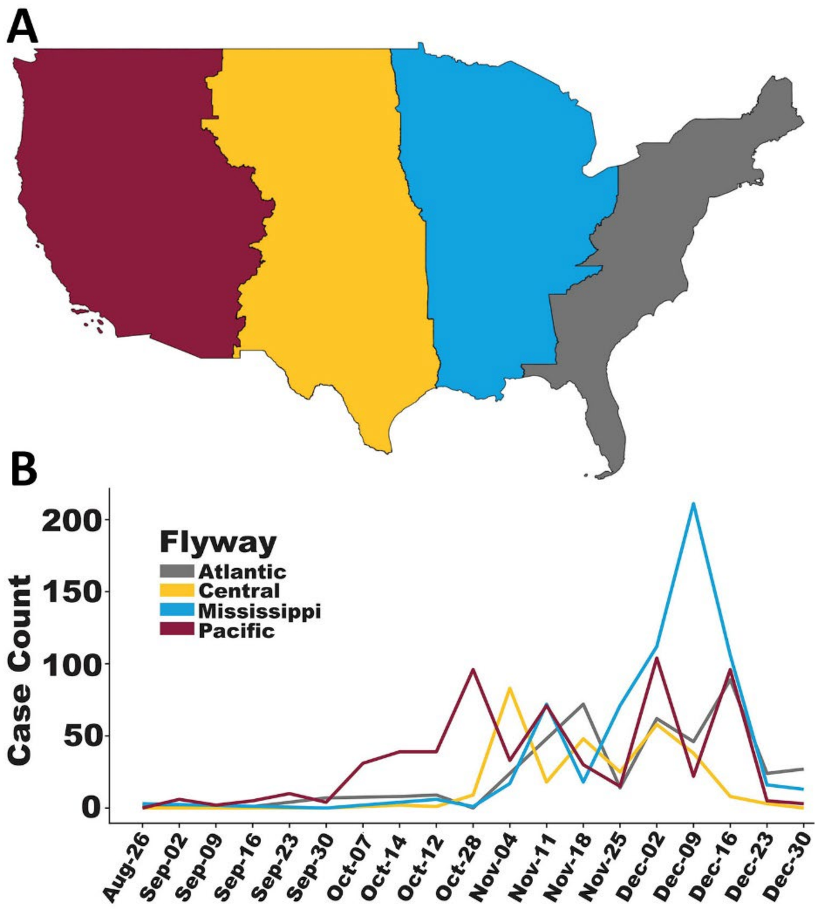

Appendix Figure 5. Avian flyway regions and confirmed HPAI case counts, United States. A) US flyways; Pacific (red), Central (yellow), Mississippi (blue), and Atlantic (gray) based on the United States Fish and Wildlife Service (*35*). B) USDA-confirmed case counts of highly pathogenic avian influenza 2.3.4.4b among wild birds by North American flyway, August 26, 2024–December 30, 2024.

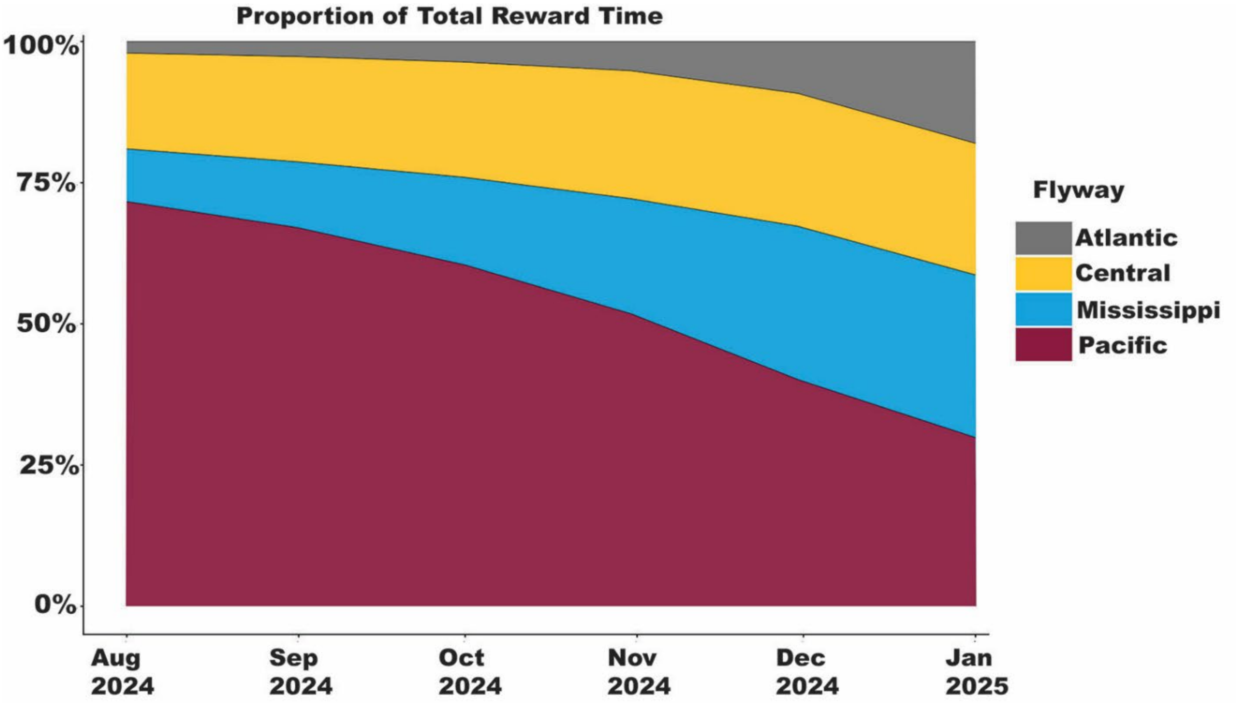

Appendix Figure 6. Proportion of total Markov rewards of D1.1 in each flyway, October 2024–March 2025.

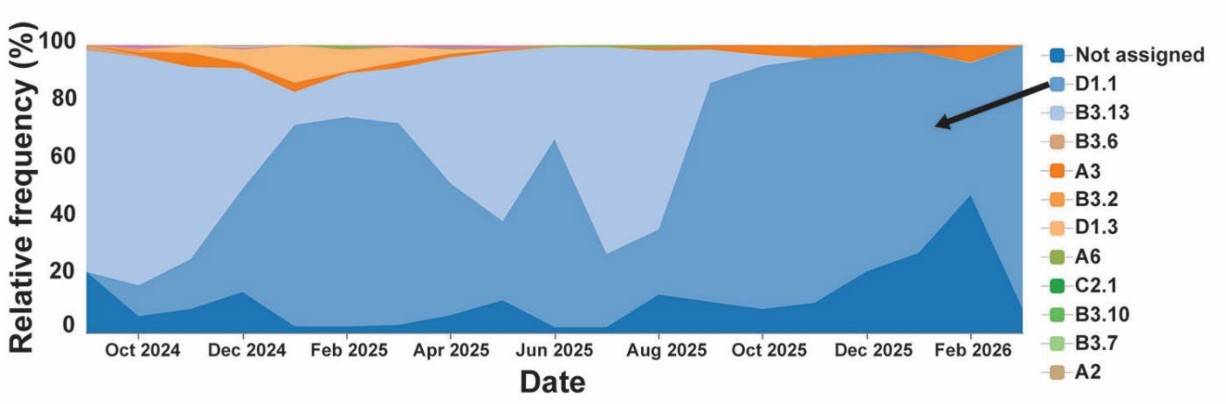

Appendix Figure 7. Relative genotype abundance in the United States of America from September 2024–March 2026 as reported on GISAID's Frequency Dashboard v0.9 (*13*). The arrow indicates D1.1.
